# [2,2′-Bis(diphenyl­phosphan­yl)-1,1′-binaphthyl-κ^2^ *P*,*P*′]chlorido(4-methyl­phenyl­sulfon­yl-κ*S*)palladium(II) dichloro­methane tris­olvate monohydrate

**DOI:** 10.1107/S1600536811049889

**Published:** 2011-11-25

**Authors:** Ruja Shrestha, William W. Brennessel, Daniel J. Weix

**Affiliations:** a Department of Chemistry, University of Rochester, Rochester, NY 14627, USA

## Abstract

In the title compound, [Pd(C_7_H_7_O_2_S)Cl(C_44_H_32_P_2_)]·3CH_2_Cl_2_·H_2_O, the geometry around the metal atom is distorted square planar, with a twist angle between the P—Pd—P and S—Pd—Cl planes of 28.11 (2)°. The two Pd—P bond lengths differ by about 0.04 Å and the biphosphane bite angle is slightly obtuse [92.92 (2)°]. There are three dichloro­methane and one water mol­ecule co-crystallized with the palladium mol­ecule, all with atoms in general positions. Alternating water and palladium mol­ecules form four-membered cyclic units through O—H⋯Cl and O—H⋯O hydrogen bonding. One of the dichloromethane solvent molecules is disordered over two positions in a 0.55:0.45 ratio.

## Related literature

For the only other structurally characterized complex with a closely related ligand set, see: Li *et al.* (2003 ▶). For the synthesis of the precursor complex (BINAP)PdCl_2_, see: Ozawa *et al.* (1993 ▶). For an additional related example with spectroscopic characterization, see: Kashiwabara & Tanaka (2005 ▶). For a description of the Cambridge Structural Database, see: Allen (2002 ▶). 
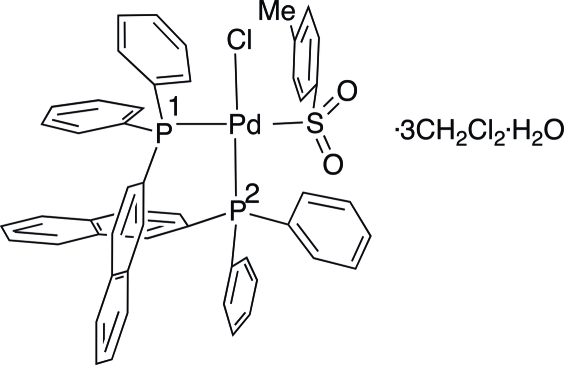


## Experimental

### 

#### Crystal data

[Pd(C_7_H_7_O_2_S)Cl(C_44_H_32_P_2_)]·3CH_2_Cl_2_·H_2_O
*M* *_r_* = 1192.47
Triclinic, 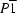

*a* = 12.7273 (12) Å
*b* = 13.7474 (13) Å
*c* = 16.533 (3) Å
α = 101.808 (2)°
β = 101.339 (2)°
γ = 109.743 (2)°
*V* = 2551.8 (5) Å^3^
*Z* = 2
Mo *K*α radiation
μ = 0.88 mm^−1^
*T* = 100 K
0.34 × 0.18 × 0.06 mm

#### Data collection

Bruker SMART APEXII CCD diffractometer
Absorption correction: multi-scan (*SADABS*; Sheldrick, 2007 ▶) *T*_min_ = 0.754, *T*_max_ = 0.949
55527 measured reflections
22145 independent reflections
15533 reflections with *I* > 2σ(*I*)
*R*_int_ = 0.051

#### Refinement

*R*[*F* ^2^ > 2σ(*F* ^2^)] = 0.051
*wR*(*F* ^2^) = 0.128
*S* = 1.03
22145 reflections
629 parameters
3 restraints
H atoms treated by a mixture of independent and constrained refinement
Δρ_max_ = 3.12 e Å^−3^
Δρ_min_ = −1.19 e Å^−3^

### 

Data collection: *APEX2* (Bruker, 2007 ▶); cell refinement: *SAINT* (Bruker, 2007 ▶); data reduction: *SAINT*; program(s) used to solve structure: *SIR97* (Altomare *et al.*, 1999 ▶); program(s) used to refine structure: *SHELXL97* (Sheldrick, 2008 ▶); molecular graphics: *SHELXTL* (Sheldrick, 2008 ▶); software used to prepare material for publication: *SHELXTL*.

## Supplementary Material

Crystal structure: contains datablock(s) I, global. DOI: 10.1107/S1600536811049889/br2184sup1.cif

Structure factors: contains datablock(s) I. DOI: 10.1107/S1600536811049889/br2184Isup2.hkl

Additional supplementary materials:  crystallographic information; 3D view; checkCIF report

## Figures and Tables

**Table 1:** Selected bond lengths (Å)

Pd1—P2	2.2574 (6)
Pd1—P1	2.2990 (7)
Pd1—S1	2.3331 (7)
Pd1—Cl1	2.3710 (6)

**Table 2:** Hydrogen-bond geometry (Å, °)

*D*—H⋯*A*	*D*—H	H⋯*A*	*D*⋯*A*	*D*—H⋯*A*
O3—H3*A*⋯Cl1^i^	0.78 (4)	2.49 (4)	3.236 (2)	159 (3)
O3—H3*B*⋯O1^ii^	0.80 (4)	2.07 (4)	2.834 (3)	162 (4)

Symmetry codes: (i) 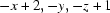; (ii) 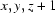.

**Fig. 1.**
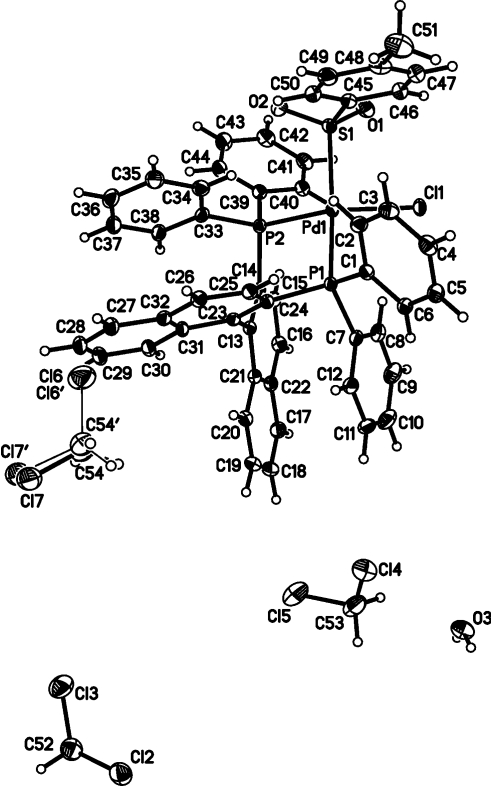
A displacement ellisoid (50% probability) drawing of the title complex.

**Fig. 2.**
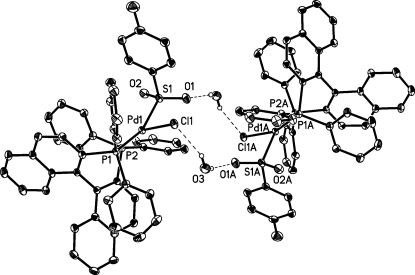
Dimers are formed via hydrogen bonding. Dichloromethane solvent molecules (which do not participate in hydrogen bonding) have been omitted for clarity.

## supplementary crystallographic information

### Comment

We report the synthesis, isolation and structural characterization of the title compound, (BINAP)Pd(SO_2_-4-methylphenyl)Cl, BINAP = 2,2'-bis(diphenylphosphano)-1,1'-binaphthyl. To date the only other structurally characterized palladium complex with a related ligand set (biphosphane, SO_2_R, halide) is (dppf)Pd(SO_2_Me)Cl, dppf = 1,1'-bis(diphenylphosphanyl)ferrocene, synthesized *via* insertion of SO_2_ into the Pd–C bond of (dppf)Pd(Me)Cl (Li *et al.*, 2003, CSD refcode WUYJUV (Allen, 2002)). The distorted square planar geometry around the metal center is typical for 16-electron palladium complexes, with the *cis* angles ranging from 88.60 (2) to 93.84 (2)° and with a twist angle between the P–Pd–P and S–Pd–Cl planes of 28.11 (2)°. The biphosphane bite angle is 92.92 (2)°. While the Pd–S and Pd–Cl bond lengths in the title compound are similar to those in (dppf)Pd(SO_2_Me)Cl, the individual and average Pd–P bond lengths are shorter in the title compound than they are in the latter complex: the average Pd–P bond lengths are 2.2782 (9) and 2.3436 (11) Å, respectively (Li *et al.*, 2003).

The asymmetric unit contains one palladium monomer, one water and three dichloromethane solvent molecules, all with atoms in general positions (Fig. 1). One dichloromethane molecule is modeled as disordered over two positions (55:45). Larger units are formed *via* hydrogen bonding among two palladium and two water molecules (Fig. 2).

### Experimental

^1^H and ^31^P{^1^H} NMR spectra were recorded on a Bruker 400 MHz spectrometer with residual protiated solvent as a reference for ^1^H NMR (δ = 7.26 ppm) and 85% H_3_PO_4_ (δ = 0.00 ppm) as a reference for ^31^P NMR. Dry solvents were prepared from ACS grade, inhibitor free solvents by passage through activated alumina and molecular sieves in a Vacuum Atmospheres solvent purification system. Water content was routinely measured using Karl-Fisher titration (Metrohm) and was less than 30 ppm in all cases. NMR solvents were purchased from Cambridge Isotope Laboratories and used after vacuum transfer from calcium hydride. PdCl_2_ (Strem Chemicals), BINAP (Alfa Aesar) and *p*-toluenesulfinate sodium salt (Aldrich) were purchased commercially and used as received.

Under an inert atmosphere, in a dry bomb flask equipped with a magnetic stir bar, 400 mg (0.5 mmol) of (BINAP)PdCl_2_ (Ozawa *et al.*, 1993), 178 mg (1.0 mmol) of *p*-tolSO_2_Na and 10 mL THF were added and stirred overnight at 333 K. The reaction was stopped when the yellow solution changed color to bright orange. The reaction mixture was filtered through celite. The filtrate was removed under reduced pressure. The bright orange residue obtained was dissolved in a minimum volume of CH_2_Cl_2_ and layered with pentane. The supernatant was removed and the bright orange crystals obtained were dried under reduced pressure. Vapor diffusion of pentane into a solution of material dissolved in CH_2_Cl_2_ afforded crystals appropriate for X-ray crystal diffraction. The crystals obtained were characterized by ^1^H, ^31^P{^1^H} NMR, IR spectroscopy and X-ray diffraction. ^1^H NMR (CDCl_3_, ppm): δ 2.45 (s, 3H), 7.2-8.3 (m, 32 H). ^31^P NMR (CDCl_3_, ppm): δ 21.4 (d, J = 45 Hz), 31.6 (d, J = 45 Hz). IR (KBr, *ν*S═O): 1265, 1088 cm^-1^. The IR data compare well to those of the (PPh3)Pd(SO_2_Ph)Cl complex (Kashiwabara and Tanaka, 2005).

### Refinement

One of the co-crystallized dichloromethane solvent molecules is modeled as disordered over two positions (55:45). Bond lengths and angles in both orientations of the disordered dichloromethane molecule were restrained to be similar. Atoms CL6 and CL6' were constrained to be isopositional. Anisotropic displacement parameters for spatially close atom pairs were constrained to be equivalent.

The hydrogen atoms of the co-crystallized water molecule were found from the difference Fourier map and refined independently from the oxygen atom with individual isotropic displacement parameters. All other hydrogen atoms were placed geometrically and refined relative to the carbon atoms for position and thermal motion (*U*_iso_[H] = 1.2**U*_eq_[*C*(non-methyl)] or 1.5**U*_eq_[*C*(methyl)]).

The maximum residual peak of 3.13 e/Å^3^ in the final difference map, located 0.71 Å from the Pd atom, is likely the result of residual absorption errors or a very minor systematic problem with the data (e.g., unresolved twinning, etc.). The maximum residual peak located away from a metal center is found 0.82 Å from atom CL5, and is likely a very minor occupancy disorder position of the chloride atom. The deepest hole of -1.16 e/Å^3^, located 0.45 Å from atom CL7', is likely the result of imperfect disorder modeling.

### Crystal data

**Table:**

[Pd(C_7_H_7_O_2_S)Cl(C_44_H_32_P_2_)]·3CH_2_Cl_2_·H_2_O	*Z* = 2
*M**_r_* = 1192.47	*F*(000) = 1212
Triclinic, *P*1	*D*_x_ = 1.552 Mg m^−^^3^
Hall symbol: -P 1	Mo *K*α radiation, λ = 0.71073 Å
*a* = 12.7273 (12) Å	Cell parameters from 9765 reflections
*b* = 13.7474 (13) Å	θ = 2.3–38.0°
*c* = 16.533 (3) Å	µ = 0.88 mm^−^^1^
α = 101.808 (2)°	*T* = 100 K
β = 101.339 (2)°	Plate, orange
γ = 109.743 (2)°	0.34 × 0.18 × 0.06 mm
*V* = 2551.8 (5) Å^3^	

### Data collection

**Table:**

Bruker SMART APEXII CCD diffractometer	22145 independent reflections
Radiation source: fine-focus sealed tube	15533 reflections with *I* > 2σ(*I*)
graphite	*R*_int_ = 0.051
area detector, ω scans at different φ	θ_max_ = 35.0°, θ_min_ = 1.7°
Absorption correction: multi-scan (*SADABS*; Sheldrick, 2007)	*h* = −20→20
*T*_min_ = 0.754, *T*_max_ = 0.949	*k* = −22→22
55527 measured reflections	*l* = −26→26

### Refinement

**Table:**

Refinement on *F*^2^	Primary atom site location: structure-invariant direct methods
Least-squares matrix: full	Secondary atom site location: difference Fourier map
*R*[*F*^2^ > 2σ(*F*^2^)] = 0.051	Hydrogen site location: inferred from neighbouring sites
*wR*(*F*^2^) = 0.128	H atoms treated by a mixture of independent and constrained refinement
*S* = 1.03	*w* = 1/[σ^2^(*F*_o_^2^) + (0.059*P*)^2^ + 0.5385*P*] where *P* = (*F*_o_^2^ + 2*F*_c_^2^)/3
22145 reflections	(Δ/σ)_max_ = 0.001
629 parameters	Δρ_max_ = 3.12 e Å^−^^3^
3 restraints	Δρ_min_ = −1.19 e Å^−^^3^

### Special details

**Table:**

Geometry. All e.s.d.'s (except the e.s.d. in the dihedral angle between two l.s. planes) are estimated using the full covariance matrix. The cell e.s.d.'s are taken into account individually in the estimation of e.s.d.'s in distances, angles and torsion angles; correlations between e.s.d.'s in cell parameters are only used when they are defined by crystal symmetry. An approximate (isotropic) treatment of cell e.s.d.'s is used for estimating e.s.d.'s involving l.s. planes.
Refinement. Refinement of *F*^2^ against ALL reflections. The weighted *R*-factor *wR* and goodness of fit *S* are based on *F*^2^, conventional *R*-factors *R* are based on *F*, with *F* set to zero for negative *F*^2^. The threshold expression of *F*^2^ > σ(*F*^2^) is used only for calculating *R*-factors(gt) *etc*. and is not relevant to the choice of reflections for refinement. *R*-factors based on *F*^2^ are statistically about twice as large as those based on *F*, and *R*- factors based on ALL data will be even larger.

### Fractional atomic coordinates and isotropic or equivalent isotropic displacement parameters (Å^2^)

**Table:**

	*x*	*y*	*z*	*U*_iso_*/*U*_eq_	Occ. (<1)
Pd1	0.984460 (14)	0.254567 (14)	0.170687 (11)	0.01342 (4)	
Cl1	1.16871 (5)	0.24732 (5)	0.22063 (4)	0.01911 (10)	
P1	0.99218 (5)	0.32202 (5)	0.31194 (4)	0.01322 (10)	
P2	0.78889 (5)	0.19958 (5)	0.12437 (4)	0.01378 (10)	
S1	1.00768 (5)	0.25072 (5)	0.03371 (4)	0.01730 (10)	
O1	1.04773 (16)	0.16952 (15)	−0.00573 (12)	0.0245 (4)	
O2	0.91331 (16)	0.26402 (16)	−0.02318 (12)	0.0240 (4)	
C1	1.13412 (19)	0.43331 (18)	0.36018 (15)	0.0159 (4)	
C2	1.1737 (2)	0.50283 (19)	0.31162 (15)	0.0191 (4)	
H2	1.1249	0.4933	0.2567	0.023*	
C3	1.2834 (2)	0.5854 (2)	0.34332 (16)	0.0215 (5)	
H3	1.3091	0.6333	0.3106	0.026*	
C4	1.3563 (2)	0.5986 (2)	0.42264 (17)	0.0227 (5)	
H4	1.4319	0.6549	0.4439	0.027*	
C5	1.3187 (2)	0.5296 (2)	0.47086 (16)	0.0223 (5)	
H5	1.3688	0.5388	0.5252	0.027*	
C6	1.2077 (2)	0.4468 (2)	0.44019 (15)	0.0186 (4)	
H6	1.1822	0.3997	0.4735	0.022*	
C7	0.97608 (19)	0.23210 (18)	0.37843 (15)	0.0166 (4)	
C8	0.9668 (2)	0.1269 (2)	0.34504 (17)	0.0213 (5)	
H8	0.9759	0.1046	0.2895	0.026*	
C9	0.9441 (2)	0.0551 (2)	0.39340 (19)	0.0275 (5)	
H9	0.9370	−0.0169	0.3705	0.033*	
C10	0.9318 (2)	0.0872 (2)	0.47488 (19)	0.0293 (6)	
H10	0.9151	0.0371	0.5073	0.035*	
C11	0.9440 (2)	0.1926 (2)	0.50894 (17)	0.0244 (5)	
H11	0.9377	0.2153	0.5654	0.029*	
C12	0.9653 (2)	0.2652 (2)	0.46091 (15)	0.0194 (4)	
H12	0.9726	0.3371	0.4841	0.023*	
C13	0.72688 (18)	0.19554 (17)	0.27927 (14)	0.0140 (4)	
C14	0.72625 (18)	0.13534 (17)	0.20028 (14)	0.0141 (4)	
C15	0.6869 (2)	0.02110 (18)	0.18069 (15)	0.0174 (4)	
H15	0.6851	−0.0200	0.1263	0.021*	
C16	0.6515 (2)	−0.03050 (19)	0.23889 (16)	0.0194 (4)	
H16	0.6262	−0.1069	0.2246	0.023*	
C17	0.6193 (2)	−0.0251 (2)	0.38152 (17)	0.0224 (5)	
H17	0.5958	−0.1013	0.3681	0.027*	
C18	0.6212 (2)	0.0321 (2)	0.46000 (18)	0.0251 (5)	
H18	0.6011	−0.0037	0.5016	0.030*	
C19	0.6531 (2)	0.1446 (2)	0.47905 (17)	0.0231 (5)	
H19	0.6517	0.1840	0.5330	0.028*	
C20	0.6860 (2)	0.1982 (2)	0.42126 (15)	0.0190 (4)	
H20	0.7082	0.2742	0.4359	0.023*	
C21	0.68735 (19)	0.14164 (18)	0.33960 (15)	0.0162 (4)	
C22	0.65194 (19)	0.02749 (19)	0.31962 (16)	0.0178 (4)	
C23	0.76806 (19)	0.31632 (17)	0.30225 (14)	0.0138 (4)	
C24	0.88568 (19)	0.38189 (18)	0.32198 (14)	0.0145 (4)	
C25	0.9210 (2)	0.49524 (18)	0.34099 (14)	0.0164 (4)	
H25	1.0015	0.5399	0.3555	0.020*	
C26	0.8414 (2)	0.54170 (18)	0.33889 (15)	0.0176 (4)	
H26	0.8671	0.6179	0.3502	0.021*	
C27	0.6375 (2)	0.5244 (2)	0.31807 (16)	0.0212 (5)	
H27	0.6618	0.6004	0.3284	0.025*	
C28	0.5224 (2)	0.4617 (2)	0.30143 (16)	0.0229 (5)	
H28	0.4671	0.4941	0.3006	0.027*	
C29	0.4852 (2)	0.3491 (2)	0.28554 (16)	0.0231 (5)	
H29	0.4048	0.3058	0.2743	0.028*	
C30	0.5637 (2)	0.30137 (19)	0.28612 (15)	0.0185 (4)	
H30	0.5372	0.2252	0.2749	0.022*	
C31	0.68385 (19)	0.36371 (18)	0.30311 (14)	0.0156 (4)	
C32	0.7215 (2)	0.47790 (18)	0.32015 (14)	0.0164 (4)	
C33	0.7248 (2)	0.29563 (19)	0.10867 (14)	0.0168 (4)	
C34	0.7952 (2)	0.39884 (19)	0.11076 (16)	0.0205 (4)	
H34	0.8765	0.4183	0.1195	0.025*	
C35	0.7469 (3)	0.4740 (2)	0.10004 (18)	0.0266 (5)	
H35	0.7950	0.5446	0.1013	0.032*	
C36	0.6281 (3)	0.4454 (2)	0.08756 (17)	0.0276 (6)	
H36	0.5950	0.4966	0.0802	0.033*	
C37	0.5577 (2)	0.3427 (2)	0.08568 (16)	0.0257 (5)	
H37	0.4765	0.3239	0.0775	0.031*	
C38	0.6052 (2)	0.2670 (2)	0.09569 (15)	0.0194 (4)	
H38	0.5566	0.1963	0.0937	0.023*	
C39	0.73753 (19)	0.09204 (18)	0.02366 (14)	0.0163 (4)	
C40	0.7939 (2)	0.02060 (19)	0.01685 (16)	0.0183 (4)	
H40	0.8523	0.0262	0.0653	0.022*	
C41	0.7651 (2)	−0.0581 (2)	−0.06017 (16)	0.0218 (5)	
H41	0.8032	−0.1067	−0.0645	0.026*	
C42	0.6804 (2)	−0.0658 (2)	−0.13095 (17)	0.0241 (5)	
H42	0.6621	−0.1186	−0.1843	0.029*	
C43	0.6224 (2)	0.0030 (2)	−0.12437 (16)	0.0244 (5)	
H43	0.5632	−0.0040	−0.1729	0.029*	
C44	0.6503 (2)	0.0826 (2)	−0.04698 (15)	0.0206 (4)	
H44	0.6103	0.1298	−0.0425	0.025*	
C45	1.1288 (2)	0.37812 (19)	0.06411 (15)	0.0185 (4)	
C46	1.2422 (2)	0.3836 (2)	0.08485 (16)	0.0211 (4)	
H46	1.2566	0.3195	0.0795	0.025*	
C47	1.3344 (2)	0.4843 (2)	0.11351 (17)	0.0232 (5)	
H47	1.4123	0.4886	0.1275	0.028*	
C48	1.3142 (2)	0.5790 (2)	0.12202 (17)	0.0250 (5)	
C49	1.1998 (2)	0.5712 (2)	0.10011 (17)	0.0242 (5)	
H49	1.1848	0.6350	0.1047	0.029*	
C50	1.1084 (2)	0.4721 (2)	0.07185 (17)	0.0222 (5)	
H50	1.0306	0.4678	0.0575	0.027*	
C51	1.4146 (3)	0.6861 (3)	0.1523 (2)	0.0404 (7)	
H51A	1.4793	0.6830	0.1937	0.061*	
H51B	1.3908	0.7418	0.1803	0.061*	
H51C	1.4398	0.7037	0.1029	0.061*	
C52	0.8141 (3)	0.7969 (3)	0.1659 (2)	0.0362 (7)	
H52A	0.8546	0.8762	0.1796	0.043*	
H52B	0.8644	0.7630	0.1441	0.043*	
Cl2	0.68188 (7)	0.75253 (7)	0.08601 (5)	0.03948 (18)	
Cl3	0.79193 (9)	0.76377 (7)	0.26067 (6)	0.0452 (2)	
C53	0.8372 (3)	0.1678 (3)	0.7730 (2)	0.0386 (7)	
H53A	0.8389	0.1822	0.8345	0.046*	
H53B	0.8603	0.1062	0.7576	0.046*	
Cl4	0.93786 (8)	0.28336 (8)	0.75821 (6)	0.0475 (2)	
Cl5	0.69207 (9)	0.13308 (8)	0.70774 (6)	0.0466 (2)	
Cl6	0.73926 (9)	0.78959 (7)	0.48139 (5)	0.0452 (2)	0.553 (2)
C54	0.7043 (6)	0.7713 (6)	0.5778 (4)	0.0381 (12)	0.553 (2)
H54A	0.7724	0.7711	0.6187	0.046*	0.553 (2)
H54B	0.6388	0.7006	0.5643	0.046*	0.553 (2)
Cl7	0.66451 (19)	0.87697 (16)	0.62695 (12)	0.0393 (3)	0.553 (2)
Cl6'	0.73926 (9)	0.78959 (7)	0.48139 (5)	0.0452 (2)	0.447 (2)
C54'	0.7452 (7)	0.8276 (8)	0.5904 (5)	0.0381 (12)	0.447 (2)
H54C	0.7591	0.7738	0.6178	0.046*	0.447 (2)
H54D	0.8103	0.8986	0.6206	0.046*	0.447 (2)
Cl7'	0.6138 (2)	0.8357 (2)	0.59866 (16)	0.0393 (3)	0.447 (2)
O3	0.9752 (2)	0.01143 (18)	0.83235 (15)	0.0305 (4)	
H3A	0.928 (3)	−0.047 (3)	0.826 (2)	0.031 (9)*	
H3B	1.011 (3)	0.057 (3)	0.877 (3)	0.043 (11)*	

### Atomic displacement parameters (Å^2^)

**Table:**

	*U*^11^	*U*^22^	*U*^33^	*U*^12^	*U*^13^	*U*^23^
Pd1	0.01213 (7)	0.01655 (7)	0.01153 (7)	0.00649 (5)	0.00305 (5)	0.00260 (5)
Cl1	0.0150 (2)	0.0249 (3)	0.0189 (2)	0.0110 (2)	0.00325 (18)	0.0052 (2)
P1	0.0124 (2)	0.0149 (2)	0.0121 (2)	0.00598 (19)	0.00271 (18)	0.00287 (19)
P2	0.0139 (2)	0.0151 (2)	0.0120 (2)	0.00681 (19)	0.00234 (19)	0.00249 (19)
S1	0.0167 (2)	0.0224 (3)	0.0140 (2)	0.0091 (2)	0.00521 (19)	0.0043 (2)
O1	0.0253 (9)	0.0250 (9)	0.0218 (9)	0.0106 (7)	0.0092 (7)	−0.0001 (7)
O2	0.0208 (8)	0.0322 (9)	0.0205 (8)	0.0115 (7)	0.0047 (7)	0.0096 (7)
C1	0.0144 (9)	0.0172 (9)	0.0154 (10)	0.0062 (8)	0.0042 (7)	0.0029 (7)
C2	0.0190 (10)	0.0209 (10)	0.0158 (10)	0.0068 (8)	0.0040 (8)	0.0046 (8)
C3	0.0217 (10)	0.0188 (10)	0.0219 (11)	0.0043 (9)	0.0079 (9)	0.0058 (9)
C4	0.0158 (10)	0.0236 (11)	0.0215 (11)	0.0040 (9)	0.0033 (8)	0.0002 (9)
C5	0.0147 (9)	0.0287 (12)	0.0183 (11)	0.0061 (9)	0.0007 (8)	0.0037 (9)
C6	0.0154 (9)	0.0237 (11)	0.0158 (10)	0.0075 (8)	0.0029 (8)	0.0051 (8)
C7	0.0142 (9)	0.0186 (10)	0.0169 (10)	0.0070 (8)	0.0023 (7)	0.0066 (8)
C8	0.0207 (10)	0.0202 (10)	0.0233 (11)	0.0100 (9)	0.0036 (9)	0.0063 (9)
C9	0.0300 (13)	0.0213 (11)	0.0314 (14)	0.0117 (10)	0.0030 (11)	0.0111 (10)
C10	0.0284 (13)	0.0301 (13)	0.0310 (14)	0.0106 (11)	0.0037 (11)	0.0185 (11)
C11	0.0208 (11)	0.0344 (13)	0.0176 (11)	0.0084 (10)	0.0044 (9)	0.0123 (10)
C12	0.0167 (9)	0.0225 (10)	0.0183 (10)	0.0072 (8)	0.0038 (8)	0.0065 (8)
C13	0.0130 (8)	0.0156 (9)	0.0140 (9)	0.0062 (7)	0.0033 (7)	0.0043 (7)
C14	0.0124 (8)	0.0166 (9)	0.0143 (9)	0.0066 (7)	0.0040 (7)	0.0044 (7)
C15	0.0172 (9)	0.0160 (9)	0.0180 (10)	0.0066 (8)	0.0047 (8)	0.0030 (8)
C16	0.0184 (10)	0.0159 (9)	0.0242 (11)	0.0068 (8)	0.0068 (9)	0.0058 (8)
C17	0.0209 (10)	0.0239 (11)	0.0276 (12)	0.0093 (9)	0.0094 (9)	0.0145 (10)
C18	0.0252 (12)	0.0344 (13)	0.0263 (13)	0.0147 (11)	0.0150 (10)	0.0181 (11)
C19	0.0253 (11)	0.0317 (13)	0.0204 (11)	0.0157 (10)	0.0125 (9)	0.0103 (10)
C20	0.0187 (10)	0.0224 (10)	0.0184 (10)	0.0107 (9)	0.0065 (8)	0.0059 (8)
C21	0.0141 (9)	0.0193 (10)	0.0166 (10)	0.0074 (8)	0.0047 (7)	0.0066 (8)
C22	0.0151 (9)	0.0197 (10)	0.0207 (11)	0.0078 (8)	0.0053 (8)	0.0080 (8)
C23	0.0160 (9)	0.0167 (9)	0.0107 (9)	0.0085 (7)	0.0042 (7)	0.0035 (7)
C24	0.0146 (9)	0.0174 (9)	0.0122 (9)	0.0077 (7)	0.0034 (7)	0.0036 (7)
C25	0.0162 (9)	0.0168 (9)	0.0150 (10)	0.0060 (8)	0.0037 (8)	0.0034 (7)
C26	0.0200 (10)	0.0167 (9)	0.0166 (10)	0.0083 (8)	0.0045 (8)	0.0044 (8)
C27	0.0244 (11)	0.0237 (11)	0.0207 (11)	0.0154 (9)	0.0072 (9)	0.0062 (9)
C28	0.0234 (11)	0.0310 (12)	0.0200 (11)	0.0185 (10)	0.0059 (9)	0.0061 (9)
C29	0.0183 (10)	0.0308 (12)	0.0214 (11)	0.0133 (9)	0.0049 (9)	0.0048 (9)
C30	0.0161 (9)	0.0207 (10)	0.0196 (11)	0.0091 (8)	0.0051 (8)	0.0044 (8)
C31	0.0151 (9)	0.0196 (10)	0.0136 (9)	0.0103 (8)	0.0033 (7)	0.0033 (7)
C32	0.0189 (9)	0.0195 (10)	0.0128 (9)	0.0105 (8)	0.0041 (8)	0.0040 (8)
C33	0.0184 (9)	0.0200 (10)	0.0128 (9)	0.0097 (8)	0.0026 (7)	0.0047 (8)
C34	0.0244 (11)	0.0197 (10)	0.0203 (11)	0.0102 (9)	0.0091 (9)	0.0071 (8)
C35	0.0349 (14)	0.0266 (12)	0.0286 (13)	0.0188 (11)	0.0149 (11)	0.0123 (10)
C36	0.0396 (15)	0.0334 (14)	0.0223 (12)	0.0270 (12)	0.0102 (11)	0.0106 (10)
C37	0.0265 (12)	0.0385 (14)	0.0183 (11)	0.0216 (11)	0.0057 (9)	0.0066 (10)
C38	0.0200 (10)	0.0236 (11)	0.0164 (10)	0.0121 (9)	0.0033 (8)	0.0047 (8)
C39	0.0154 (9)	0.0174 (9)	0.0139 (9)	0.0059 (8)	0.0031 (7)	0.0018 (7)
C40	0.0168 (9)	0.0188 (10)	0.0187 (10)	0.0070 (8)	0.0051 (8)	0.0041 (8)
C41	0.0211 (10)	0.0191 (10)	0.0232 (12)	0.0071 (9)	0.0084 (9)	0.0019 (9)
C42	0.0216 (11)	0.0230 (11)	0.0186 (11)	0.0049 (9)	0.0034 (9)	−0.0039 (9)
C43	0.0193 (10)	0.0293 (12)	0.0171 (11)	0.0079 (9)	−0.0008 (9)	−0.0005 (9)
C44	0.0170 (10)	0.0251 (11)	0.0167 (10)	0.0089 (9)	0.0021 (8)	0.0012 (8)
C45	0.0197 (10)	0.0228 (10)	0.0162 (10)	0.0102 (9)	0.0073 (8)	0.0067 (8)
C46	0.0200 (10)	0.0254 (11)	0.0217 (11)	0.0115 (9)	0.0076 (9)	0.0086 (9)
C47	0.0207 (11)	0.0269 (12)	0.0244 (12)	0.0100 (9)	0.0076 (9)	0.0096 (10)
C48	0.0291 (12)	0.0240 (11)	0.0220 (12)	0.0068 (10)	0.0118 (10)	0.0090 (9)
C49	0.0317 (13)	0.0240 (11)	0.0247 (12)	0.0154 (10)	0.0138 (10)	0.0096 (9)
C50	0.0238 (11)	0.0286 (12)	0.0216 (11)	0.0151 (10)	0.0097 (9)	0.0112 (9)
C51	0.0398 (17)	0.0318 (15)	0.0430 (18)	0.0057 (13)	0.0163 (14)	0.0080 (13)
C52	0.0323 (14)	0.0372 (16)	0.0397 (17)	0.0119 (13)	0.0122 (13)	0.0137 (13)
Cl2	0.0384 (4)	0.0423 (4)	0.0327 (4)	0.0181 (3)	0.0068 (3)	−0.0005 (3)
Cl3	0.0573 (5)	0.0453 (4)	0.0440 (5)	0.0247 (4)	0.0166 (4)	0.0253 (4)
C53	0.056 (2)	0.0401 (16)	0.0255 (14)	0.0281 (16)	0.0060 (13)	0.0103 (12)
Cl4	0.0502 (5)	0.0647 (6)	0.0435 (5)	0.0307 (4)	0.0237 (4)	0.0243 (4)
Cl5	0.0543 (5)	0.0491 (5)	0.0316 (4)	0.0198 (4)	0.0004 (4)	0.0130 (3)
Cl6	0.0676 (6)	0.0487 (5)	0.0301 (4)	0.0341 (4)	0.0169 (4)	0.0115 (3)
C54	0.037 (3)	0.051 (3)	0.027 (2)	0.021 (3)	0.006 (2)	0.011 (3)
Cl7	0.0555 (10)	0.0419 (9)	0.0355 (8)	0.0298 (7)	0.0203 (7)	0.0163 (6)
Cl6'	0.0676 (6)	0.0487 (5)	0.0301 (4)	0.0341 (4)	0.0169 (4)	0.0115 (3)
C54'	0.037 (3)	0.051 (3)	0.027 (2)	0.021 (3)	0.006 (2)	0.011 (3)
Cl7'	0.0555 (10)	0.0419 (9)	0.0355 (8)	0.0298 (7)	0.0203 (7)	0.0163 (6)
O3	0.0340 (11)	0.0235 (10)	0.0307 (11)	0.0067 (9)	0.0145 (9)	0.0032 (9)

### Geometric parameters (Å, °)

**Table:**

Pd1—P2	2.2574 (6)	C27—H27	0.9500
Pd1—P1	2.2990 (7)	C28—C29	1.406 (4)
Pd1—S1	2.3331 (7)	C28—H28	0.9500
Pd1—Cl1	2.3710 (6)	C29—C30	1.369 (3)
P1—C7	1.803 (2)	C29—H29	0.9500
P1—C1	1.819 (2)	C30—C31	1.414 (3)
P1—C24	1.824 (2)	C30—H30	0.9500
P2—C33	1.806 (2)	C31—C32	1.425 (3)
P2—C39	1.816 (2)	C33—C34	1.388 (3)
P2—C14	1.834 (2)	C33—C38	1.395 (3)
S1—O2	1.4609 (18)	C34—C35	1.393 (3)
S1—O1	1.4663 (18)	C34—H34	0.9500
S1—C45	1.791 (2)	C35—C36	1.387 (4)
C1—C6	1.399 (3)	C35—H35	0.9500
C1—C2	1.400 (3)	C36—C37	1.383 (4)
C2—C3	1.382 (3)	C36—H36	0.9500
C2—H2	0.9500	C37—C38	1.391 (3)
C3—C4	1.386 (3)	C37—H37	0.9500
C3—H3	0.9500	C38—H38	0.9500
C4—C5	1.384 (4)	C39—C44	1.394 (3)
C4—H4	0.9500	C39—C40	1.399 (3)
C5—C6	1.394 (3)	C40—C41	1.382 (3)
C5—H5	0.9500	C40—H40	0.9500
C6—H6	0.9500	C41—C42	1.384 (4)
C7—C8	1.393 (3)	C41—H41	0.9500
C7—C12	1.395 (3)	C42—C43	1.383 (4)
C8—C9	1.385 (4)	C42—H42	0.9500
C8—H8	0.9500	C43—C44	1.395 (3)
C9—C10	1.386 (4)	C43—H43	0.9500
C9—H9	0.9500	C44—H44	0.9500
C10—C11	1.385 (4)	C45—C50	1.387 (3)
C10—H10	0.9500	C45—C46	1.389 (3)
C11—C12	1.388 (3)	C46—C47	1.390 (4)
C11—H11	0.9500	C46—H46	0.9500
C12—H12	0.9500	C47—C48	1.395 (4)
C13—C14	1.392 (3)	C47—H47	0.9500
C13—C21	1.431 (3)	C48—C49	1.392 (4)
C13—C23	1.500 (3)	C48—C51	1.491 (4)
C14—C15	1.421 (3)	C49—C50	1.372 (4)
C15—C16	1.365 (3)	C49—H49	0.9500
C15—H15	0.9500	C50—H50	0.9500
C16—C22	1.406 (3)	C51—H51A	0.9800
C16—H16	0.9500	C51—H51B	0.9800
C17—C18	1.364 (4)	C51—H51C	0.9800
C17—C22	1.418 (3)	C52—Cl2	1.747 (3)
C17—H17	0.9500	C52—Cl3	1.766 (3)
C18—C19	1.408 (4)	C52—H52A	0.9900
C18—H18	0.9500	C52—H52B	0.9900
C19—C20	1.367 (3)	C53—Cl4	1.769 (4)
C19—H19	0.9500	C53—Cl5	1.792 (3)
C20—C21	1.419 (3)	C53—H53A	0.9900
C20—H20	0.9500	C53—H53B	0.9900
C21—C22	1.423 (3)	Cl6—C54	1.782 (6)
C23—C24	1.391 (3)	C54—Cl7	1.788 (6)
C23—C31	1.431 (3)	C54—H54A	0.9900
C24—C25	1.414 (3)	C54—H54B	0.9900
C25—C26	1.367 (3)	C54'—Cl7'	1.741 (8)
C25—H25	0.9500	C54'—H54C	0.9900
C26—C32	1.412 (3)	C54'—H54D	0.9900
C26—H26	0.9500	O3—H3A	0.78 (4)
C27—C28	1.363 (4)	O3—H3B	0.80 (4)
C27—C32	1.418 (3)		
			
P2—Pd1—P1	92.92 (2)	C32—C26—H26	119.6
P2—Pd1—S1	93.84 (2)	C28—C27—C32	120.9 (2)
P1—Pd1—S1	159.89 (2)	C28—C27—H27	119.5
P2—Pd1—Cl1	159.39 (2)	C32—C27—H27	119.5
P1—Pd1—Cl1	88.60 (2)	C27—C28—C29	120.1 (2)
S1—Pd1—Cl1	91.67 (2)	C27—C28—H28	120.0
C7—P1—C1	108.21 (10)	C29—C28—H28	120.0
C7—P1—C24	106.06 (10)	C30—C29—C28	120.6 (2)
C1—P1—C24	105.61 (10)	C30—C29—H29	119.7
C7—P1—Pd1	118.23 (8)	C28—C29—H29	119.7
C1—P1—Pd1	105.45 (8)	C29—C30—C31	121.0 (2)
C24—P1—Pd1	112.55 (7)	C29—C30—H30	119.5
C33—P2—C39	108.31 (10)	C31—C30—H30	119.5
C33—P2—C14	106.74 (10)	C30—C31—C32	118.37 (19)
C39—P2—C14	105.27 (10)	C30—C31—C23	122.5 (2)
C33—P2—Pd1	120.21 (8)	C32—C31—C23	119.1 (2)
C39—P2—Pd1	107.15 (7)	C26—C32—C27	121.8 (2)
C14—P2—Pd1	108.24 (7)	C26—C32—C31	119.15 (19)
O2—S1—O1	115.97 (11)	C27—C32—C31	119.1 (2)
O2—S1—C45	104.74 (11)	C34—C33—C38	120.0 (2)
O1—S1—C45	105.94 (11)	C34—C33—P2	119.65 (18)
O2—S1—Pd1	112.78 (8)	C38—C33—P2	120.36 (18)
O1—S1—Pd1	116.91 (8)	C33—C34—C35	120.1 (2)
C45—S1—Pd1	97.59 (8)	C33—C34—H34	120.0
C6—C1—C2	119.3 (2)	C35—C34—H34	120.0
C6—C1—P1	122.67 (18)	C36—C35—C34	119.7 (3)
C2—C1—P1	117.86 (17)	C36—C35—H35	120.1
C3—C2—C1	120.3 (2)	C34—C35—H35	120.1
C3—C2—H2	119.9	C37—C36—C35	120.4 (2)
C1—C2—H2	119.9	C37—C36—H36	119.8
C2—C3—C4	120.3 (2)	C35—C36—H36	119.8
C2—C3—H3	119.9	C36—C37—C38	120.2 (2)
C4—C3—H3	119.9	C36—C37—H37	119.9
C5—C4—C3	120.0 (2)	C38—C37—H37	119.9
C5—C4—H4	120.0	C37—C38—C33	119.6 (2)
C3—C4—H4	120.0	C37—C38—H38	120.2
C4—C5—C6	120.3 (2)	C33—C38—H38	120.2
C4—C5—H5	119.8	C44—C39—C40	119.9 (2)
C6—C5—H5	119.8	C44—C39—P2	122.74 (17)
C5—C6—C1	119.8 (2)	C40—C39—P2	117.27 (16)
C5—C6—H6	120.1	C41—C40—C39	120.2 (2)
C1—C6—H6	120.1	C41—C40—H40	119.9
C8—C7—C12	120.0 (2)	C39—C40—H40	119.9
C8—C7—P1	119.69 (18)	C40—C41—C42	119.8 (2)
C12—C7—P1	120.21 (17)	C40—C41—H41	120.1
C9—C8—C7	119.6 (2)	C42—C41—H41	120.1
C9—C8—H8	120.2	C43—C42—C41	120.4 (2)
C7—C8—H8	120.2	C43—C42—H42	119.8
C8—C9—C10	120.6 (2)	C41—C42—H42	119.8
C8—C9—H9	119.7	C42—C43—C44	120.5 (2)
C10—C9—H9	119.7	C42—C43—H43	119.8
C11—C10—C9	119.8 (2)	C44—C43—H43	119.8
C11—C10—H10	120.1	C39—C44—C43	119.2 (2)
C9—C10—H10	120.1	C39—C44—H44	120.4
C10—C11—C12	120.3 (2)	C43—C44—H44	120.4
C10—C11—H11	119.9	C50—C45—C46	120.1 (2)
C12—C11—H11	119.9	C50—C45—S1	119.32 (18)
C11—C12—C7	119.7 (2)	C46—C45—S1	120.44 (18)
C11—C12—H12	120.1	C45—C46—C47	119.1 (2)
C7—C12—H12	120.1	C45—C46—H46	120.4
C14—C13—C21	119.65 (19)	C47—C46—H46	120.4
C14—C13—C23	121.15 (19)	C46—C47—C48	120.9 (2)
C21—C13—C23	119.20 (19)	C46—C47—H47	119.5
C13—C14—C15	119.6 (2)	C48—C47—H47	119.5
C13—C14—P2	122.00 (16)	C49—C48—C47	118.8 (2)
C15—C14—P2	118.05 (16)	C49—C48—C51	121.2 (3)
C16—C15—C14	120.9 (2)	C47—C48—C51	120.0 (3)
C16—C15—H15	119.5	C50—C49—C48	120.5 (2)
C14—C15—H15	119.5	C50—C49—H49	119.7
C15—C16—C22	121.1 (2)	C48—C49—H49	119.7
C15—C16—H16	119.4	C49—C50—C45	120.5 (2)
C22—C16—H16	119.4	C49—C50—H50	119.8
C18—C17—C22	120.9 (2)	C45—C50—H50	119.8
C18—C17—H17	119.6	C48—C51—H51A	109.5
C22—C17—H17	119.6	C48—C51—H51B	109.5
C17—C18—C19	119.6 (2)	H51A—C51—H51B	109.5
C17—C18—H18	120.2	C48—C51—H51C	109.5
C19—C18—H18	120.2	H51A—C51—H51C	109.5
C20—C19—C18	121.0 (2)	H51B—C51—H51C	109.5
C20—C19—H19	119.5	Cl2—C52—Cl3	111.26 (17)
C18—C19—H19	119.5	Cl2—C52—H52A	109.4
C19—C20—C21	120.9 (2)	Cl3—C52—H52A	109.4
C19—C20—H20	119.5	Cl2—C52—H52B	109.4
C21—C20—H20	119.5	Cl3—C52—H52B	109.4
C20—C21—C22	117.9 (2)	H52A—C52—H52B	108.0
C20—C21—C13	122.5 (2)	Cl4—C53—Cl5	111.35 (17)
C22—C21—C13	119.6 (2)	Cl4—C53—H53A	109.4
C16—C22—C17	121.4 (2)	Cl5—C53—H53A	109.4
C16—C22—C21	119.0 (2)	Cl4—C53—H53B	109.4
C17—C22—C21	119.6 (2)	Cl5—C53—H53B	109.4
C24—C23—C31	120.03 (19)	H53A—C53—H53B	108.0
C24—C23—C13	121.13 (18)	Cl6—C54—Cl7	110.8 (4)
C31—C23—C13	118.83 (19)	Cl6—C54—H54A	109.5
C23—C24—C25	119.63 (19)	Cl7—C54—H54A	109.5
C23—C24—P1	120.38 (16)	Cl6—C54—H54B	109.5
C25—C24—P1	119.65 (16)	Cl7—C54—H54B	109.5
C26—C25—C24	121.2 (2)	H54A—C54—H54B	108.1
C26—C25—H25	119.4	Cl7'—C54'—H54C	109.8
C24—C25—H25	119.4	Cl7'—C54'—H54D	109.8
C25—C26—C32	120.8 (2)	H54C—C54'—H54D	108.3
C25—C26—H26	119.6	H3A—O3—H3B	124 (4)

### Hydrogen-bond geometry (Å, °)

**Table:**

*D*—H···*A*	*D*—H	H···*A*	*D*···*A*	*D*—H···*A*
O3—H3A···Cl1^i^	0.78 (4)	2.49 (4)	3.236 (2)	159 (3)
O3—H3B···O1^ii^	0.80 (4)	2.07 (4)	2.834 (3)	162 (4)

Symmetry codes: (i) −*x*+2, −*y*, −*z*+1; (ii) *x*, *y*, *z*+1.
